# Differential structural and resting state connectivity between insular subdivisions and other pain-related brain regions

**DOI:** 10.1016/j.pain.2014.07.009

**Published:** 2014-10

**Authors:** K. Wiech, S. Jbabdi, C.S. Lin, J. Andersson, I. Tracey

**Affiliations:** a Oxford Centre for Functional Magnetic Resonance Imaging of the Brain, University of Oxford, John Radcliffe Hospital, Headley Way, Oxford OX3 9DU, England, UK; b Nuffield Department of Clinical Neurosciences, Nuffield Division Anaesthetics, University of Oxford, John Radcliffe Hospital, Headley Way, Oxford OX3 9DU, England, UK

**Keywords:** Connectivity, Insula, Pain, Resting state, Structural

## Abstract

Functional neuroimaging studies suggest that the anterior, mid, and posterior division of the insula subserve different functions in the perception of pain. The anterior insula (AI) has predominantly been associated with cognitive–affective aspects of pain, while the mid and posterior divisions have been implicated in sensory-discriminative processing. We examined whether this functional segregation is paralleled by differences in (1) structural and (2) resting state connectivity and (3) in correlations with pain-relevant psychological traits. Analyses were restricted to the 3 insular subdivisions and other pain-related brain regions. Both type of analyses revealed largely overlapping results. The AI division was predominantly connected to the ventrolateral prefrontal cortex (structural and resting state connectivity) and orbitofrontal cortex (structural connectivity). In contrast, the posterior insula showed strong connections to the primary somatosensory cortex (SI; structural connectivity) and secondary somatosensory cortex (SII; structural and resting state connectivity). The mid insula displayed a hybrid connectivity pattern with strong connections with the ventrolateral prefrontal cortex, SII (structural and resting state connectivity) and SI (structural connectivity). Moreover, resting state connectivity revealed strong connectivity of all 3 subdivisions with the thalamus. On the behavioural level, AI structural connectivity was related to the individual degree of pain vigilance and awareness that showed a positive correlation with AI-amygdala connectivity and a negative correlation with AI–rostral anterior cingulate cortex connectivity. In sum, our findings show a differential structural and resting state connectivity for the anterior, mid, and posterior insula with other pain-relevant brain regions, which might at least partly explain their different functional profiles in pain processing.

## Summary

The anterior, mid, and posterior insula show differential structural and resting state connectivity with other pain-related brain regions.

## 1 Introduction

Of the various brain regions that have been implicated in the perception of pain, the insula has most consistently been reported across studies [17]. Insular activation has been found during acute [9], [26], [41], [62] and chronic pain [47]. Although often referred to as the insula or the insular cortex, studies in primates indicate that this structure consists of an anterior, mid, and posterior part, which differ considerably in anatomy and cytoarchitecture [5]. In accordance with these findings, functional neuroimaging studies on pain (and other sensory experiences) in humans suggest a functional segregation (see [14] for review). Activity in the posterior (PI) and mid insula (MI) predominantly reflects sensory aspects of pain [1], [35], [44]. The anterior insula (AI), in contrast, has been associated with the cognitive–affective dimension of pain that is sensitive to contextual manipulations [40], [62].

Recent findings from functional connectivity studies support the notion of a functional segregation. During noxious stimulation, PI exhibits increased functional connectivity with the primary somatosensory cortex [36] that is pivotal for the processing of sensory-discriminative information. AI, in contrast, showed increased connectivity with the prefrontal and anterior cingulate cortex [36] known to be involved in evaluative processing. Moreover, increased functional coupling between the left AI and the midcingulate cortex that has also been implicated in emotional salience monitoring [48], [56] precedes the perception of ambiguous stimuli (ie, stimuli of an intensity calibrated at the pain detection threshold) as painful [62].

Tracer studies in primates have shown direct white matter connections between the AI and prefrontal regions whereas the MI/PI is most strongly connected to somatosensory regions [5], [29], [31]. So far, explorative diffusion magnetic resonance imaging (MRI) tractography studies in humans have confirmed the dissociation between AI and MI/PI connections to prefrontal cortex and postcentral gyrus [12], [13]. However, a more comprehensive analysis on connections with the different brain regions that have been implicated in pain processing is still missing.

Here, we compared both structural and resting state connectivity of the 3 insular subdivisions with pain-related brain regions in healthy volunteers. More specifically, we used probabilistic tracto-graphy [8] to (1) identify pain-related brain regions that are predominantly connected to the 3 subdivisions and (2) identify the main insular connection target for each pain-related region. Furthermore, we (3) searched for the pain-related brain regions that showed the strongest resting state connectivity for each insular subdivision and (4) identified the part of the insula each pain-related region exhibited the strongest resting state connectivity with.

## 2 Methods

The study focuses on 2 different aspects of the interaction of the 3 insular subdivisions with other pain-related brain regions: structural connectivity and resting state connectivity. Because the 2 aspects were investigated in different samples and required different analyses, they are described separately below.

### 2.1 Structural connectivity (probabilistic tractography)

*Participants.* Sixteen healthy right-handed volunteers participated in the study. Data from 1 subject suffered technical failure and were consequently excluded; therefore, data from 15 subjects were entered into the analysis (mean ± SD age 20.6 ± 3.6, 11 women). All participants gave written informed consent. The study was approved by the Oxford Research Ethics Committee.

*Diffusion imaging and preprocessing.* The diffusion-weighted images were acquired using echo planar imaging (EPI) with a voxel size of 2 × 2 × 2 mm. The diffusion weighting was isotropically distributed along 60 directions using a b-value of 1500 s mm^−2^. Two sets of diffusion-weighted data sets were acquired in total for subsequent averaging, to improve the signal-to-noise ratio. A dual-echo blip-reversed sequence was used to reduce EPI distortions [4]. In brief, for each set, non-diffusion-weighted (b0) images with opposite phase-encode directions (blip up/down) were combined to estimate field inhomogeneities using the TOPUP tool (part of FSL5). Then the 2 averages were corrected for EPI distortions (using the calculated field), as well as eddy currents and motion, using the EDDY_CORRECT tool in FSL. The images obtained using this method had virtually no residual EPI-induced distortions, which generally are particularly pronounced in the frontal lobes.

*Probabilistic tractography.* Probabilistic tractography was performed using the FMRIB’s Diffusion Toolbox (FDT, http://fsl.fmrib.ox.ac.uk/fsl/fdt). We first used FLIRT [22], [23] for linear image transformation, then FNIRT [2], [3] for nonlinear image transformation between the diffusion and the individual structural space and between the individual structural and MNI152 standard space. This transformation was used throughout all the tractography analyses, and tractography results were directly resampled in standard space. We performed probabilistic tracking to calculate the number of streamline samples starting from any location of the brain passing through a combined region of interest mask, which consists of a seed mask and a target mask. Therefore, any found tract was constrained by the seed mask at one end and the target mask at the other. This way of sampling has been shown to increase robustness in identifying long connections and connections that traverse regions with multiple crossing fibers, such as the connections between the insular and cingulate cortex [51]. Note that this approach differs from those starting from voxels within the seed mask only but uses a standard algorithm as described in [7]. To quantify the uncertainty of tracking results, we drew 100 sample streamlines for each voxel in the brain. The output file was split into a seed-to-target map, which revealed how many streamline samples can successfully reach the target region from each voxel in the seed region. For each insular subdivision, 24 tractography analyses were performed for the 12 targets, separately in both hemispheres. Only ipsilateral connections (eg, between the right AI to the right VLPFC) were analyzed. To exclude indirect tracts (ie, tracts that pass through more than one pain-related region before reaching the target region), we used a stop mask at the target region and used the other targets as exclusion masks.

*Psychological assessment.* All participants filled in the following questionnaires to assess pain-related psychological traits: (1) Center for Epidemiological Studies Depression Scale (CES-D) [43], (2) State and Trait Anxiety Inventory [52], (3) Pain Vigilance and Awareness Questionnaire (PVAQ) [27], and (4) the Pain Catastrophizing Scale [54]. All questionnaires were analyzed according to their respective manual.

### 2.2 Resting state connectivity

We used resting state data from a cohort of healthy subjects that participated in previous studies conducted in our laboratory [50]. Note that this sample did not overlap with the sample in which the structural connectivity was investigated.

*Participants.* Resting state data sets were acquired from 36 healthy adult subjects (age range, 20–35 years; mean, 28.5 years; 15 women). The study was approved by the institutional review board and subjects provided informed consent.

*Data acquisition.* Resting fMRI blood oxygenation level dependent (BOLD) data were acquired on a 3 T Siemens Trio MRI scanner, using a 12-channel head coil with a standard gradient echo echo-planar-imaging (EPI) acquisition, TR = 2 s, TE = 28 ms, flip angle = 89°, resolution 3 × 3 × 3.5 mm, whole-head coverage except for the lowest parts of the cerebellum in some subjects. The resting fMRI scan lasted 6 min. Ambient light was minimized, and the subjects were instructed to lie with eyes open, think of nothing in particular, and not to fall asleep. In order to aid the registration of the functional data into a common standard brain coordinate system (MNI152), structural brain images were acquired by using a T1-weighted 3--D MPRAGE sequence with whole-head coverage, TR = 2.04 s, TE = 4.7 ms, flip angle = 8° resolution 1 × 1 × 1 mm, total acquisition time 12 min.

### 2.3 Preparation of masks

**Fig. 1:**
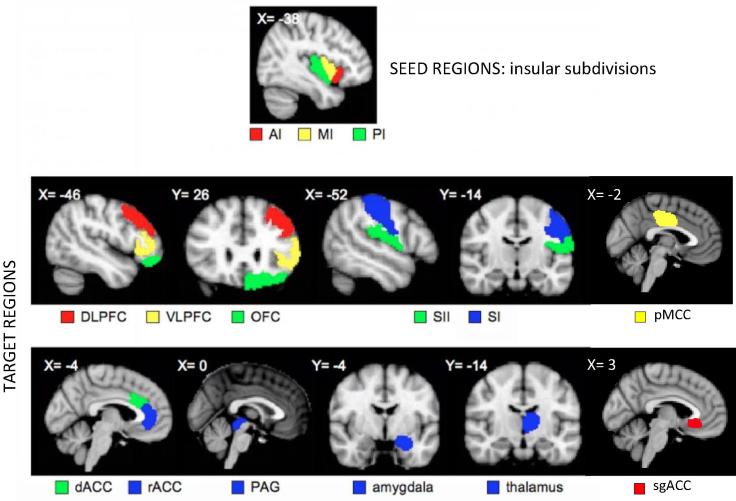
Seed and target regions of interest for probabilistic tractography. Seeds comprise 3 insular subdivisions: anterior (AI), mid (MI), and posterior (PI). Targets comprise 12 pain-related targets: thalamus (THA), primary and secondary somatosensory cortex (SI and SII), dorsal and rostral anterior cingulate cortex (dACC and rACC), dorsolateral and ventrolateral prefrontal cortex (DLPFC and VLPFC), amygdala (AMYG), orbitofrontal cortex (OFC), periaqueductal grey (PAG), posterior midcingulate cortex (pMCC), and subgenual anterior cingulate cortex (sgACC). Note that same regions of interest were used in analyses on resting state functional connectivity.

The same set of anatomically defined masks was used in the analysis of structural, and resting state connectivity. Thirty masks of 3 bilateral seed (ie, AI, MI, and PI) and 12 pain-related target regions of interest were defined according to published atlases and manual drawings based on individual T1-weighted images (Fig. 1). Pain-related target regions comprised the thalamus (THA), primary and secondary somatosensory cortices (SI and SII), the subgenual, dorsal, and rostral anterior cingulate cortex (sgACC, dACC, and rACC), posterior midcingulate cortex (pMCC), dorsolateral and ventrolateral prefrontal cortex (DLPFC and VLPFC), amygdala (AMYG), orbitofrontal cortex (OFC), and periaqueductal grey (PAG). The THA and the AMYG were selected from the Harvard-Oxford Subcortical Atlas (Harvard Center for Morphometric Analysis), thresholded at 25%. SI and SII were selected from the Juelich Histological Atlas (The Research Center Juelich), thresholded at 25%. The masks of the sgACC, dACC, rACC, pMCC, DLPFC, VLPFC, AMYG, OFC, PAG, and the 3 subdivisions of the insula were defined on each subject’s T1-weighted image and hand drawn using fslview (in FSL, FMRIB Software Library, http://www.fmrib.ox.ac.uk/fsl) starting in one view, eg, coronal view, and double-checked using multiplanar views.

*Anterior, mid, and posterior insula.* The insula was divided into anterior, mid, and posterior regions as defined by Brooks et al. [9]. The mask for the AI includes tissue from the anterior gyrus brevis, the MI comprises the posterior gyrus brevis, and the PI was defined as the anterior gyrus longus.

*Dorsal and rostral anterior cingulate cortex.* The mask for the dACC corresponds to the anterior midcingulate cortex (Brodmann area 24′ and 32′), while the rACC was defined as the perigenual ACC (area 24 and 32) following the division proposed by Vogt et al. [58].

*Subgenual anterior cingulate cortex.* The subgenual ACC is located underneath the genu of the corpus callosum and corresponds mainly to BA25 and caudal portions of BA32 and BA24. The mask was drawn in sagittal view, starting from the inferior border of rACC and moving posteriorly to the end of the genu.

*Dorsolateral and ventrolateral prefrontal cortex.* The mask for the DLPFC comprises BA8, 9, 46, and 9/46 located in the superior and middle frontal gyrus. VLPFC was defined as Brodmann areas 44 (pars opercularis), 45 (pars triangularis), and the lateral part of area 47/12 of the inferior frontal gyrus.

*Orbitofrontal cortex.* The OFC mask comprises Brodmann areas 10, 11, 12, 13, 14, and the orbital part of area 47/12. It extends from the horizontal ramus of the lateral fissure on the lateral surface to the orbital surface and onto the medial surface to include the gyrus ventral to cingulate sulcus and the subcallosal cingulate areas. The boundary on the medial surface is from the rostral sulcus to the horizontal ramus of the lateral fissure.

*Periaqueductal grey.* The outline of PAG was first delineated in the sagittal view, and the mask was drawn in the axial view according to the Duvernoy’s atlas of the human brain stem [32].

*Posterior midcingulate cortex.* The pMCC is located between the corpus callosum inferiorly and the cingulate sulcus superiorly. The posterior border is the continuation of the central sulcus, while the anterior border is defined by the sulcus midway between its posterior border and the posterior border of the dorsal ACC. The mask was outlined in sagittal view and filled up in coronal view.

### 2.4 Data analysis

#### 2.4.1 Structural connectivity

*Definition of connection probability indices.* In the following analyses, we refer to connection probabilities as the number of streamlines from probabilistic tractography that satisfy a given condition (eg, connect to the AI), divided by the total number of streamlines seeded that were not rejected by the exclusion criteria. This probability reflects our uncertainty on the fiber orientation measurements and the tractography process. In analyses 1 and 2, we identify the dominant connection targets of each insular subdivision (analysis 1) and the dominant insular connection target for each pain-related brain region (analysis 2), separately for both hemispheres. In these analyses, we report relative target probabilities that represent the probability that a tract reaches a particular target region, relative to all targets (in %).

*Analysis 1: Main pain-related connection targets for each insular subdivision.* In order to identify the dominant connection targets for each insular subdivision, we performed 2-way ANOVAs with the factors HEMISPHERE (left, right) and TARGET (sgACC, rACC, dACC, pMCC, SI, SII, THA, PAG, OFC, AMYG, DLPFC, and VLPFC) on the relative target probability.

*Analysis 2: Main insular connection target for each pain-related brain region.* In this analysis, we compared the structural connectivity with the 3 insular subdivisions, separately for each pain-related brain region using a 2-way ANOVA with the factors HEMISPHERE (left, right) and SUBDIVISION (AI, MI, PI).

*Analysis 3: Overall structural connectivity of the insular subdivisions with pain-related brain regions (across all target regions).* For each insular subdivision in both hemispheres, we first averaged the seed-to-target probabilities for all 12 ipsilateral targets to calculate the mean seed-to-target probability for each subdivision. Subsequently, we performed a repeated-measures ANOVA with the factors HEMISPHERE (left, right) and SUBDIVISION (AI, MI, PI) on these indices.

*Analysis 4: Correlation with pain-relevant psychological traits.* To test whether the structural connectivity between the insular subdivisions and target regions is related to pain-relevant psychological traits, Pearson correlations were calculated between the relative connectivity strength of each connection (averaged across both hemispheres) and the questionnaire scores (corrected for multiple comparisons).

#### 2.4.2 Resting state connectivity

Data preprocessing was carried out with FSL tools. The following prestatistics processing was applied for each subject: head motion correction by using MCFLIRT [22]; nonbrain removal by using BET [49]; spatial smoothing by using a Gaussian kernel of FWHM 5 mm; grand-mean intensity normalization of the entire 4-D data set by a single multiplicative factor; high-pass temporal filtering (subtraction of Gaussian-weighted least-squares straight-line fitting, with sigma = 50.0 s). Registration of each subject’s fMRI data to that subject’s high-resolution structural image was carried out by using FLIRT [22]. Registration from the high-resolution structurals to MNI152 standard space was achieved by using FLIRT affine registration and then further refined by using FNIRT nonlinear registration [2], [3].

All subjects’ 4-D fMRI time series data were transformed into standard space at 2 × 2 × 2 mm resolution using the registration transformations derived as described above. For each subject, functional connectivity between insular subdivisions and other pain-related regions was calculated using the fsl_sbca tool (as described in [33]). Briefly, the first Eigen-time-series of each of the pain-related regions were calculated; conditional correlations between the 3 insular subdivisions and the pain-related brain regions were then calculated. To this end, the Eigen-time-series of any 2 out of the 3 insular subregions were regressed out from the third subregion and the pain-related brain regions before calculating the correlation coefficient. The result is a set of partial correlation coefficients that determine interdependence between insula time series and other pain regions that are not shared across the insular subregions.

*Analysis 5: Identification of differences in resting state connectivity between targets.* The conditional correlation coefficients we calculated were entered into a 2-way ANOVA with the factors HEMISPHERE (left, right) and TARGET (sgACC, rACC, dACC, pMCC, SI, SII, THA, PAG, OFC, AMYG, DLPFC, and VLPFC) separately for each of the 3 insular subdivisions.

*Analysis 6: Identification of differences in resting state connectivity for each pain-related brain regions with the 3 insular subdivisions.* Conditional correlation coefficients characterizing the resting state connectivity were entered into a 2-way ANOVA with the factors HEMISPHERE (left, right) and SUBDIVISION (AI, MI, PI).

Significant ANOVA results were followed up by post hoc *t* test which were adjusted for multiple comparisons using Bonferroni correction. Results reaching a *P* value of <.05 were considered statistically significant. To increase the readability of the results for analyses 2 and 5, test statistics and *P* values are provided in Tables S1 and S2.

## 3 Results

### 3.1 Structural connectivity

**Fig. 2:**
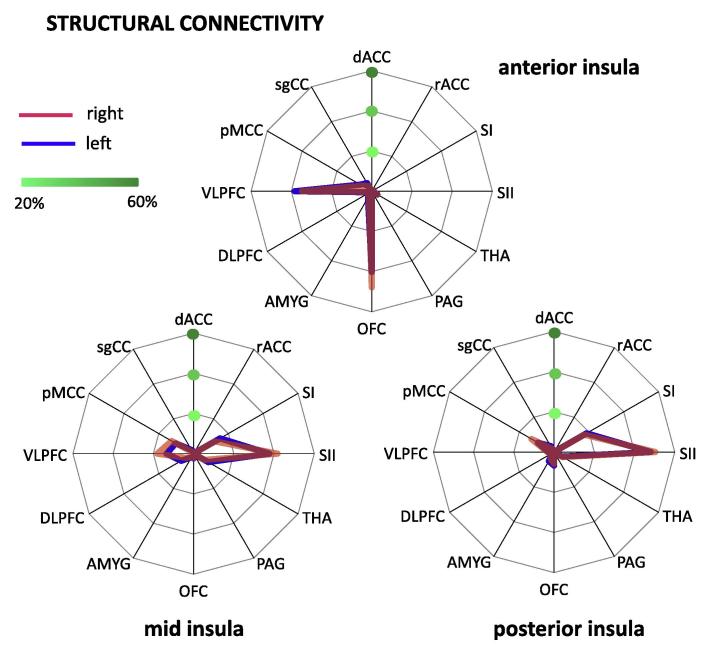
Differential structural connectivity of insular subdivisions with pain-related brain regions. (A) Anterior insula showed significantly higher connection probability with orbitofrontal cortex (OFC) and ventrolateral prefrontal cortex (VLPFC) than other pain-related regions. (B) Mid insula showed higher connection probability with primary (SI) and secondary (SII) somatosensory cortex, VLPFC, and pMCC compared to other pain-related regions. (C) Posterior insula was preferentially connected to SI, SII, and pMCC. dACC, dorsal anterior cingulate cortex; rACC, rostral anterior cingulate cortex; SI, primary somatosensory cortex; SII, secondary somatosensory cortex; THA, thalamus; PAG, periaqueductal grey; OFC, orbitofrontal cortex; AMYG, amygdala; DLPFC, dorsolateral prefrontal cortex; VLPFC, ventrolateral prefrontal cortex; pMCC, posterior midcingulate cortex; sgACC, subgenual anterior cingulate cortex.

*Analysis 1: Main connection targets of each insular subdivision.* The analysis for the AI showed a main effect of TARGET (*F*(1.94,27.12) = 178.89, *P* < .001; Fig. 2), indicating that this subdivision was differentially connected with the various pain-related brain regions. Pairwise comparisons between targets revealed a significantly higher connection probability with the OFC (left: 40.2%, right: 47.9.1%) and VLPFC (left: 38.3.4%, right: 34.7%) compared to the other targets. The difference between OFC and VLPFC only reached statistical significance for the right hemisphere with stronger connectivity of the OFC (*t*(14) = 5.02, *P* < .001). Furthermore, the AI showed a significant interaction between TARGET and HEMISPHERE (*F*(1.95,27.31) = 8.27, *P* = .002), suggesting that the differential connectivity with pain-related brain regions varied between hemispheres. Pairwise comparisons between hemispheres revealed that the AI is more strongly connected with the OFC in the right than the left hemisphere (*t*(14) = 5.53, *P* = .001). All other comparisons did not reach significance (ie, *P* > .05).

The MI showed a main effect of TARGET (*F*(2.11,29.51) = 55.81, *P* < *P*.001) (Fig. 2). The highest connection probability was found for SII (left: 37.7%, right: 41.8%), followed by the VLPFC (left: 13.5%, right: 18.0%), SI (left: 15.2%, right: 12.2%) and pMCC (left: 10.3%, right: 12.6%). The connection probability for SII was significantly higher than for any other target. The MI also exhibited a significant interaction between TARGET and HEMISPHERE (MI: (*F*(3.21,44.96) = 4.12, *P* = .01). However, pairwise comparisons revealed no difference in connection probability between hemispheres when corrected for multiple comparisons.

The PI showed a main effect of TARGET (*F*(1.78,24.95) = 56.10, *P* < .001) (Fig. 2). The highest connection probability was for SII (left: 48.1%, right: 50.3%), followed by SI (left: 18.6%, right: 17.3%) and the pMCC (left: 9.3%, right: 18.1%). The probability for SII was significantly higher than for any other target. In contrast to the AI and MI, we found no interaction between HEMISPHERE and TARGET (*P* > .05) for the posterior subdivision.

**Fig. 3:**
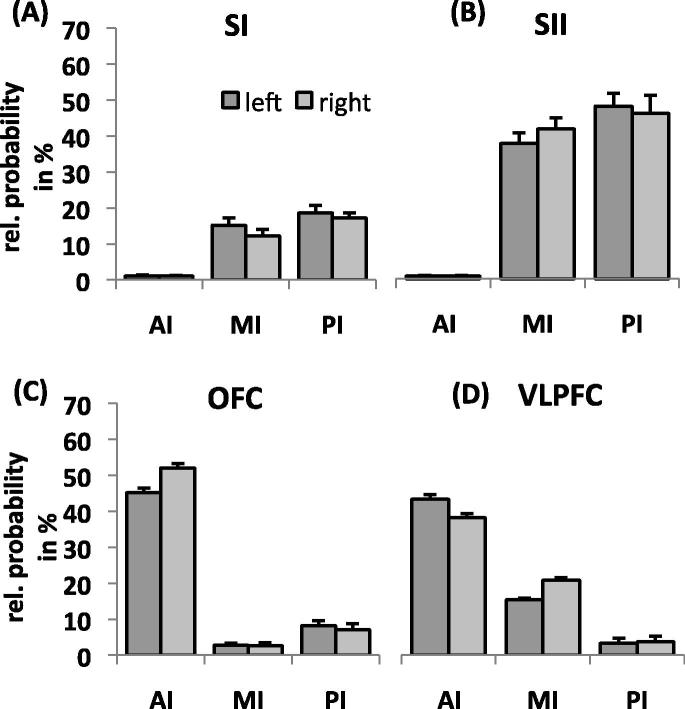
Structural connectivity of main connection targets with insular subdivisions. Primary (A) and secondary (B) somatosensory cortices were more strongly connected to mid (MI) and posterior insula (PI) than anterior insula (AI). In contrast, orbitofrontal cortex (C) and ventrolateral prefrontal cortex (D) were preferentially connected with AI.

*Analysis 2: Main insular connection targets of each pain-related brain region.* As revealed by a 2-way ANOVA, the structural connectivity of the rACC, AMYG, and DLPFC with the insula (across subdivisions) was more pronounced in the left than the right hemisphere (main effect HEMISPHERE). In contrast, the OFC showed a higher connectivity for the right than the left side. A main effect of SUBDIVISION, indicating differential structural connectivity with the 3 insular subdivisions was found for all pain-related brain regions, except the ACC targets (ie, dACC, rACC, and sgACC). A posterior–anterior gradient in connectivity (ie, decrease in connectivity from PI to AI) was found for SI, SII, pMCC, and the PAG. An anterior–posterior gradient (ie, a decrease in connectivity from AI to PI) was revealed for the VLPFC. The OFC and amygdala exhibited stronger connectivity with both AI and PI relative to MI. The DLPFC and thalamus showed a preference for MI relative to AI and PI. Figure 3 displays the connectivity pattern of the target regions that showed the strongest differential connectivity in Analysis 1 (ie, SI, SII, OFC, and VLPFC). Details on significant interactions are provided in Table S1.

**Fig. 4:**
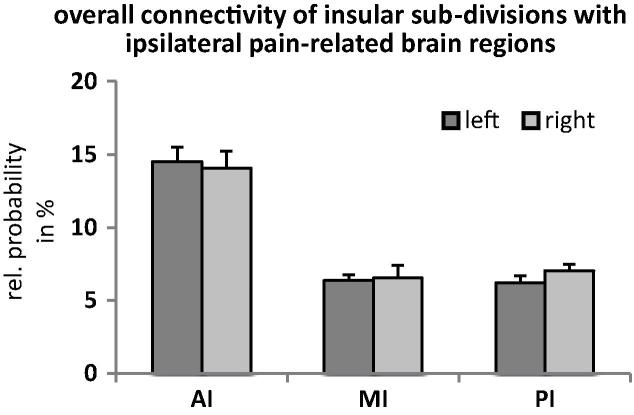
Overall structural connectivity of 3 insular subdivisions with ipsilateral pain-related brain regions. Structural connectivity of the anterior insula (AI) across all ipsilateral pain-related brain regions was significantly higher than the connectivity of the mid (MI) and posterior insula (PI).

*Analysis 3: Overall structural connectivity of the insular subdivisions with pain-related brain regions (across all target regions).* The 2-way ANOVA revealed a significant main effect of SUBDIVISION on the mean seed-to-target probabilities (*F*(1.26,17.65) = 65.06, *P* < .001; Fig. 4). Pairwise comparisons between the subdivisions showed that the AI had a significantly higher connection probability than the MI (*t*(14) = 8.29, *P* < .001) and PI (*t*(14) = 8.53, *P* < .001). The connection probabilities for the MI and PI were not significantly different (*P* > .5). The effect of HEMISPHERE and the interaction between the 2 factors did not reach statistical significance (both *P* > .05).

**Fig. 5:**
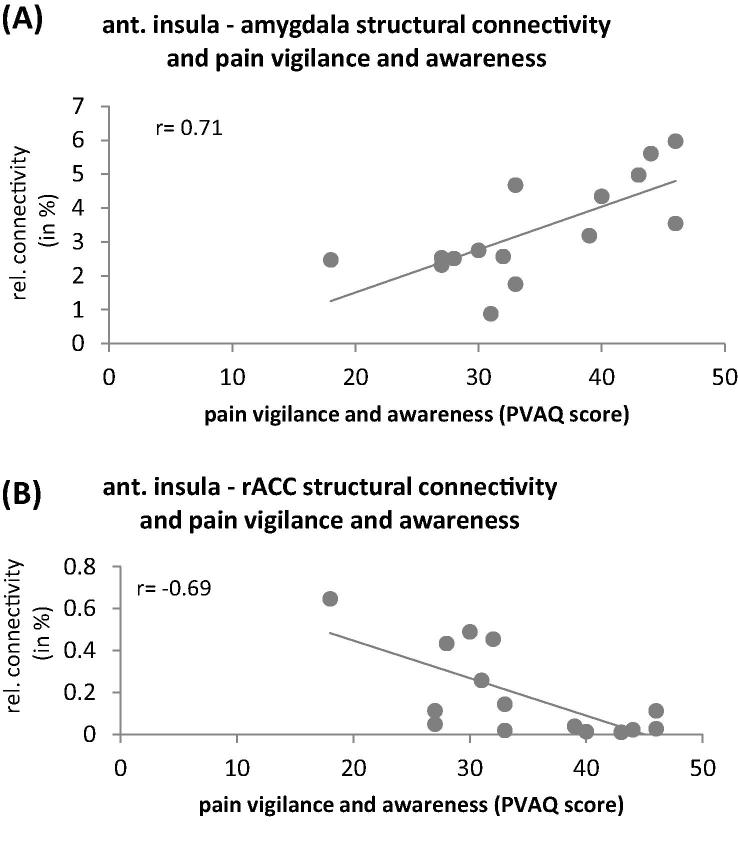
Correlation between structural connectivity of anterior insula (AI) (averaged across both hemispheres) and pain vigilance and awareness. Pain vigilance and awareness as a relevant pain-related psychological trait is positively correlated with structural connectivity between AI and amygdala (A) and negatively correlated with connectivity between AI and rostral anterior cingulate cortex (B).

*Analysis 4: Correlation with pain-relevant psychological traits.* The correlation analyses revealed that pain vigilance and awareness was positively related to the structural connectivity between the AI and amygdala (*r* = 0.71, *P* = .04; Fig. 5A) and negatively related to the connectivity between AI and rACC (*r* = −0.69; *P* = .05; Fig. 5B).

### 3.2 Resting state connectivity

**Fig. 6:**
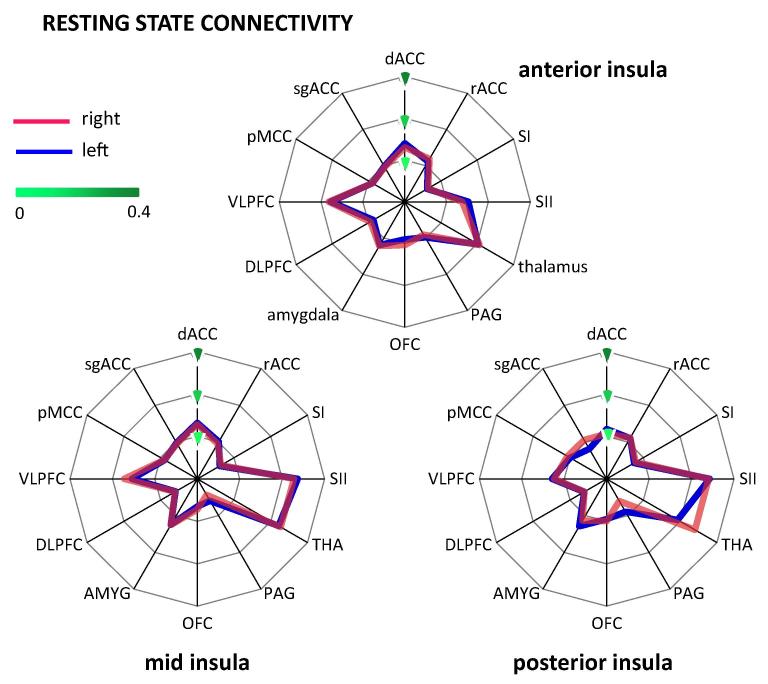
Resting state connectivity of each insular subdivision with pain-related brain regions. (A) Anterior insula showed strongest resting state connectivity with the thalamus, followed by VLPFC. (B) For the mid insula, strongest results were found for the thalamus, SII, and VLPFC. (C) The posterior insula was most strongly connected to SII and the thalamus. dACC, dorsal anterior cingulate cortex; rACC, rostral anterior cingulate cortex; SI, primary somatosensory cortex; SII, secondary somatosensory cortex; THA, thalamus; PAG, periaqueductal grey; OFC, orbitofrontal cortex; AMYG, amygdala; DLPFC, dorsolateral prefrontal cortex; VLPFC, ventrolateral prefrontal cortex; pMCC, posterior midcingulate cortex; sgACC, subgenual anterior cingulate cortex.

*Analysis 5: Main connection target of each insular subdivision.* Resting state connectivity of the AI showed a significant main effect of REGION (*F*(6.70,234.59) = 35.54, *P* < .001; Fig. 6; Table S2). Post hoc tests revealed that across hemispheres, the AI was most strongly connected to the thalamus, followed by the VLPFC (*t*(35) = 2.21, *P* = .034) and SII (*t*(35) = 3.77; *P* = .001). We found no significant main effect for HEMISPHERE (*F*(1,35) = 0.22, *P* = .644) or the interaction between REGION and HEMISPHERE (*F*(7.17,250.96) = 1.15, *P* = .329).

For MI, the resting state connectivity differed significantly between regions (main effect REGION (*F*(7.31,255.96) = 97.31, *P* < .001); Fig. 6), whereas the main effect of HEMISPHERE (*F*(1,35) = 0.02; *P* = .885) and the interaction (*F*(6.93,242.69) = 1.81, *P* = .088) were not significant. As revealed by post hoc tests across hemispheres, MI showed the strongest resting state connectivity with the thalamus and SII, which did not differ significantly (*t*(35) = 0.83, *P* = .414). The connectivity with these 2 targets was significantly stronger than with the VLPFC (comparison with THA: *t*(35) = 6.94, *P* < .001; SII: *t*(35) = 7.28, *P* < .001).

In contrast, the PI showed a main effect of REGION (*F*(7.31,255.53) = 111.69; *P* < .001; Fig. 6). Post hoc tests across hemispheres revealed that PI was most strongly connected with SII and the thalamus with significantly weaker connectivity for the latter (*t*(35) = 3.11, *P* = .004). Furthermore we found a significant interaction between REGION and HEMISPHERE (*F*(7.06,247.15) = 7.37, *P* < .001). Post hoc tests showed a side difference only for the thalamus with a stronger resting state connectivity with PI on the right side (*t*(35) = 6.14; *P* < .001). The main effect of HEMISPHERE was not significant (*F*(1,35) = 0.04; *P* = .850).

**Fig. 7:**
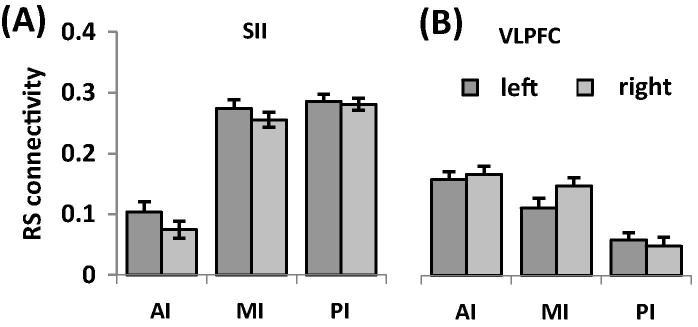
Resting state connectivity of insular subdivisions with pain-related brain regions. (A) Secondary somatosensory cortex (SII) showed stronger resting state connectivity with mid and posterior insula compared to anterior insula (AI). (B) In contrast, ventrolateral prefrontal cortex (VLPFC) exhibited stronger connectivity with AI than with posterior insula.

*Analysis 6: Main insular connection targets of each pain-related brain region.* Of the 12 pain-related brain regions, the thalamus, PAG, and pMCC showed a significant main effect of HEMISPHERE with stronger resting state connectivity on the right for the thalamus and pMCC and on the left for the PAG (Table S2). A main effect of SUBDIVISION was found for all pain-related brain regions, except the thalamus, amygdala, pMCC, and sgACC, indicating that most pain-related regions show a different functional connectivity with the 3 insular divisions during rest. Post hoc tests revealed an anterior–posterior gradient in connectivity in the dACC, VLPFC and DLPFC (Fig. 7). A posterior–anterior gradient was found in SI and SII (Fig. 7). The rACC, PAG, and OFC showed stronger connectivity in AI and PI than in MI. A significant interaction between both factors was found in the thalamus, VLPFC, and sgACC. For the thalamus, the stronger connectivity with AI relative to PI was more pronounced in the left hemisphere while the connectivity for PI than MI was significantly higher on the right side. For the VLPFC, the difference between PI and MI was stronger on the left than the right side. Post hoc tests for the sgACC did not reveal any significant differences after correction for multiple comparisons.

## 4 Discussion

In this study, we investigated the differential structural and resting state connectivity of the AI, MI, and PI with other pain-related brain regions. The analyses revealed largely overlapping findings for the 2 types of connectivity. AI was predominantly connected to (pre-)frontal brain regions, namely the OFC (structural connectivity) and VLPFC (structural and resting state connectivity). PI showed the strongest connectivity with SI (structural connectivity) and SII (structural and resting state connectivity). MI displayed a hybrid pattern of connectivity with dominant connections to the VLPFC, SII (structural and resting state connectivity) and SI (structural connectivity).

### 4.1 AI and (pre-)frontal cortex

Our observation that AI is predominantly connected to the OFC and VLPFC confirms recent evidence on direct connections between the insula and both (pre-)frontal regions [13]. Functionally, AI activation has been implicated in a number of tasks and contexts involving affective processing (see [25] for review), including pain [9], [26], [41], [62]. AI activation has been found when participants are awaiting an aversive outcome [39], [41], [62] and the level of activation has been shown to scale with subsequent pain avoidance behavior [55]. It has therefore been argued that activation in the AI represents a ‘global emotional moment’ [45] that reflects the net evaluation of the affective impact of an impending situation. In response to salient events, the AI can gate subsequent processing by activating a cognitive control network including the DLPFC and posterior parietal cortex [53]. Our data indicate that the AI is anatomically well situated to also influence affective processing through its connections with key regions involved in evaluative processing (ie, OFC) and emotion regulation (ie, VLPFC).

Like the AI, the OFC and VLPFC are both sensitive to emotional stimuli. However, the OFC primarily responds to the reward value of the stimulus (including negative value) rather than its sensory features. Importantly, OFC responses also encode the anticipation of future outcome [24], which makes it well suited for guiding subsequent decisions.

The VLPFC plays a key role in modulating the impact of painful and other emotionally relevant stimuli on behavior and subjective emotional states [30]. Activation in this brain region has been associated with voluntary attenuation of emotions [34] and suppression of negative emotions [38]. In the context of pain, VLPFC activation has been found during perceived control over pain [46], [61], placebo analgesia [37], and belief-related pain modulation [60]. It has recently been argued that emotion regulation strategies might be implemented via the interplay between VLPFC and AI [30]. In line with this notion, patients with bipolar disorder seem to lack the negative correlation between VLPFC and right AI that was found in healthy controls during performance of a cognitive interference task [42]. Moreover, voluntary control of AI activation using real-time fMRI feedback leads to concomitant changes in VLPFC activity [57] and increased functional connectivity between both regions has been found during noxious stimulation [36]. On the basis of these findings, Menon and Uddin postulated that the core function of the AI is to detect relevant stimuli and engage executive brain regions (such as the VLPFC) to ensure an appropriate response [28]. The strong anatomical connections between AI and OFC as well as VLPFC may therefore underlie the critical role of the anterior division in cognitive–affective pain modulation.

The link between the AI and psychological pain processing is further corroborated by our correlation analyses with PVAQ scores reflecting the disposition to attend to pain. This disposition is known to scale with pain-related anxiety [27], is related to a heightened perception of acute pain and is a relevant predictor for the development of chronic pain. High PVAQ scores were seen in those participants with stronger connectivity between AI and the amygdala (Fig. 5A). This result is in accordance with the recent observation of a positive correlation between AI-amygdala structural connectivity and trait anxiety in healthy volunteers [6].

PVAQ scores were also negatively correlated with the degree of structural connectivity between AI and the rACC (Fig. 5B), which is - together with the PAG - a key component of the descending pain inhibitory pathway. Although speculative at this stage, a weak relative structural connectivity between AI and rACC might be related to only limited access to this pain-attenuating pathway even though the AI might have detected a painful event as highly salient. In accordance with this interpretation, Ploner et al. reported a negative correlation between PVAQ scores and the functional connectivity between AI and PAG [41].

A rather surprising finding was the low probability for structural connections between AI and the dorsal anterior cingulate cortex (dACC). Several studies have reported increased functional connectivity between both regions in the context of pain [62] and other emotional experiences [48]. Furthermore, there is evidence for direct anatomical connections from a diffusion tensor imaging (DTI) study in humans [20], although this study did not differentiate between subdivisions of the insula and ACC. Besides an actual weak connectivity, our result might have been confounded by the problem of resolving crossing fibers [59]. Connections between the insula and ACC must pass through several large white matter tracts (eg, cortico-spinal, superior longitudinal fascicle, and corpus callosum). We could not resolve such complex fiber architecture with the data used in this study. However, we only report relative connection probabilities that reflect connectivity relative to other targets. Recent advances in diffusion MRI acquisition and preprocessing, as part of the Human Connectome Project initiative [19], may enable more robust tracking of insula–ACC connections (see [51]).

### 4.2 PI and somatosensory cortices

PI showed strongest connections with SII in both the structural and resting state connectivity and with SI in the structural connectivity analysis (Fig. 2, Fig. 6). Our findings thereby confirm observations from tracer studies in macaques [29], [31], previous DTI studies in humans [12], and resting state analyses [11], [16]. Together with lateral thalamic nuclei and PI, both somatosensory regions constitute the lateral pain system that conveys sensory-discriminatory aspects of pain. Activation in the PI reflects the transition from nonpainful to painful sensations with increasing stimulus intensity [35] and is specific for noxious relative to innocuous stimulation [26]. The link between PI (and MI) and sensory-discriminative processing is further corroborated by the moderately strong structural connectivity with the pMCC where nociceptive neurons have been identified in vivo [21] and ex vivo [18].

Given the evidence from animal tracer studies for direct connections between the thalamus and PI [15], the low connection probability for the thalamus in our analysis on structural connectivity is surprising. Information about the physiological state of the body is projected from lamina 1 of the spinal cord to the ventromedial thalamic nucleus, which in turn projects to the MI/PI [15]. It has, however, been pointed out that direct inferences from these animal data to structural connectivity in humans are difficult because the insula has undergone considerable expansion in humans [14] and the anatomy varies significantly across species [10].

### 4.3 Hybrid connectivity pattern of the MI

The structural connectivity of the MI has previously been reported in connection with the results for PI [12], [13], despite the cytoarchitectonic differences between both subdivisions [29], [31]. However, our findings show minor but potentially relevant differences. In addition to the somatosensory cortices that are also key targets of PI, the MI is strongly connected to the VLPFC (Fig. 2, Fig. 4). It is therefore the only insular subdivision that has strong connections with sensory-discriminative (ie, SI and SII) as well as to a cognitive–affective brain region (ie, the VLPFC). Widespread connections of MI have previously been reported in a whole-brain DTI study [12] where MI showed connections with parietal and temporal regions, the inferior frontal gyrus, OFC and premotor cortex. Together, this finding of a hybrid connectivity pattern [13] of MI is in line with the notion that this region integrates sensory and cognitive–emotional information [14].

### 4.4 Conclusion

Taken together, our data indicate that AI, MI, and PI are differentially connected with pain-related brain regions and that their connectivity is linked to pain-relevant behaviour. On the basis of these findings, it is tempting to speculate that these differences account for the differential functional role of the insular subdivisions in the perception of pain and beyond. Given that the insula is a multimodal region and that it is the most consistently activated brain region across all neuroimaging studies [63], our findings are equally relevant for other domains. Studies combining further behavioural indicators with functional and structural neuroimaging measures will aid in unraveling the functional significance of our findings. Finally, future studies should explore whether interindividual differences in behavioural and functional pain processing are indeed paralleled by differences in structural and resting state connectivity within distinct subsystems of the pain processing system. A more detailed understanding of the relationship between structure, function and behaviour promises insights into the resilience and susceptibility to conditions such as chronic pain.

## Conflict of interest

The authors report no conflict of interest.

## Appendix A Supplementary data

Supplementary data 1 This document file contains Supplementary Materials.
