# Supplementary material for: Differential structural and resting state connectivity between insular subdivisions and other pain-related brain regions
Source: Pain. 2014 Oct;155(10):2047–55. doi: 10.1016/j.pain.2014.07.009 (PMC4220010; doi:10.1016/j.pain.2014.07.009)
Supplement: Supplementary data 1 — This document file contains Supplementary Materials. [file mmc1.doc]

**Supplementary Material**

**for**

**Differential structural and resting state connectivity between insular sub-divisions and other pain-related brain regions**

Wiech, K., Jbabdi, S., Lin, C.S., Andersson, J., Tracey, I.

**Supplementary Results**

**Table S1: Structural connectivity (probabilistic tractography; Analysis 2**)

| **pain-related region** | **main effect**  **HEMISPHERE** | | | **main effect**  **insular SUBDIVISION** | | | **interaction** | | |
| --- | --- | --- | --- | --- | --- | --- | --- | --- | --- |
|  | **F** | **p** | **post-hoc** | **F** | **p** | **post-hoc** | **F** | **p** | **post-hoc** |
| dACC | 0.35 | 0.564 | - | 4.41 | 0.054 | - | 0.12 | 0.771 | - |
| rACC | 6.87 | 0.020 | L>R  (p= 0.020) | 2.23 | 0.140 | - | 0.05 | 0.858 | - |
| SI | 2.29 | 0.152 | - | 65.44 | < 0.001 | MI>AI***  PI>AI***  PI>MI* | 1.53 | 0.237 | - |
| SII | 0.46 | 0.51 | - | 132.85 | < 0.001 | MI>AI***  PI>AI***  PI>MI** | 2.08 | 0.163 | - |
| thalamus | 2.39 | 0.145 | - | 28.59 | < 0.001 | MI>AI***  PI>AI***  MI>PI** | 5.87 | 0.015 | - |
| PAG | 5.31 | 0.037 | - | 14.89 | < 0.001 | MI>AI**  PI>AI** | 1.43 | 0.257 | - |
| OFC | 9.63 | 0.008 | R>L  (p= 0.020) | 397.08 | < 0.001 | AI>MI***  AI>PI***  PI>MI*** | 14.57 | <0.001 | R>L(AI>MI)**  R>L(AI>PI)** |
| amygdala | 13.52 | 0.002 | L>R  (p= 0.003) | 23.29 | < 0.001 | AI>MI**  PI>AI*  PI>MI*** | 6.78 | 0.011 | L>R(PI>MI)*** |
| DLPFC | 7.20 | 0.018 | L>R  (p= 0.019) | 37.88 | < 0.001 | MI>AI***  MI>PI*** | 7.39 | 0.016 | L>R(MI>AI)* |
| VLPFC | 0.21 | 0.653 | - | 472.72 | < 0.001 | AI>MI***  AI>PI***  MI>PI*** | 11.65 | < 0.001 | L>R(AI>MI)***  R>L(MI>PI)* |
| pMCC | 1.36 | 0.263 | - | 4.97 | 0.028 | MI>AI*** | 1.89 | 0.186 | - |
| sgACC | 4.50 | 0.052 | - | 3.54 | 0.069 | - | 1.03 | 0.346 | - |

***: p<0.001; **: p< 0.01; *: p<0.05; dACC= dorsal anterior cingulate cortex; rACC= rostral anterior cingulate cortex; SI= primary somatosensory cortex; SII= secondary somatosensory cortex; PAG= periaqueductal gray; OFC= orbitofrontal cortex; DLPFC= dorsolateral prefrontal cortex; VLPFC= ventrolateral prefrontal cortex; pMCC= posterior mid cingulate cortex; sgACC= subgenual anterior cingulate cortex

**Table S2: Resting state connectivity (Analysis 6)**

| **pain-related region** | **main effect**  **HEMISPHERE** | | | **main effect**  **insular SUBDIVISION** | | | **interaction** | | |
| --- | --- | --- | --- | --- | --- | --- | --- | --- | --- |
|  | **F** | **p** | **post-hoc** | **F** | **p** | **post-hoc** | **F** | **p** | **post-hoc** |
| dACC | 3.20 | 0.082 | - | 8.41 | 0.001 | AI>PI**  MI>PI*** | 0.86 | 0.426 | - |
| rACC | 0.02 | 0.896 | - | 7.33 | 0.001 | AI>MI**  PI>MI** | 1.79 | 0.175 | - |
| SI | 0.40 | 0.531 | - | 4.77 | 0.011 | PI>MI* | 0.045 | 0.96 | - |
| SII | 3.22 | 0.082 | - | 234.89 | <0.001 | MI>AI***  PI>AI*** | 2.03 | 0.139 | - |
| thalamus | 11.32 | 0.002 | R>L  (p= 0.002**) | 4.04 | 0.02 | - | 18.34 | <0.001 | R>L(PI>AI)**  R>L(PI>MI)** |
| PAG | 9.23 | 0.004 | L> R  (p= 0.004**) | 26.77 | <0.001 | AI>MI***  AI>PI*  PI>MI*** | 2.28 | 0.110 | - |
| OFC | 0.96 | 0.333 | - | 14.40 | <0.001 | AI>MI***  PI>MI*** | 0.80 | 0.452 | - |
| amygdala | 0.76 | 0.391 | - | 0.52 | 0.60 | - | 2.91 | 0.060 | - |
| DLPFC | 0.21 | 0.647 | - | 33.60 | <0.001 | AI>MI***  AI>PI*** | 1.14 | 0.327 | - |
| VLPFC | 0.87 | 0.357 | - | 63.28 | <0.001 | AI>MI***  AI>PI***  MI>PI*** | 5.22 | 0.008 | R>L(MI>PI)** |
| pMCC | 5.14 | 0.04 | R>L  (p=0.030)* | 0.26 | 0.68 | - | 2.70 | 0.082 | - |
| sgACC | 2.93 | 0.10 | - | 1.90 | 0.163 | - | 3.14 | 0.069 | - |

***: p<0.001; **: p< 0.01; *: p<0.05; dACC= dorsal anterior cingulate cortex; rACC= rostral anterior cingulate cortex; SI= primary somatosensory cortex; SII= secondary somatosensory cortex; PAG= periaqueductal gray; OFC= orbitofrontal cortex; DLPFC= dorsolateral prefrontal cortex; VLPFC= ventrolateral prefrontal cortex; pMCC= posterior mid cingulate cortex; sgACC= subgenual anterior cingulate cortex
